# Mechanical Ball Milling-Assisted Synthesis of Esterified Starch for Polybutylene Succinate Blend with Improved Performance

**DOI:** 10.3390/molecules30204088

**Published:** 2025-10-15

**Authors:** Wenjing Cai, Canqi Huo, Jisuan Tan, Zirun Chen, Yanzhen Yin, Yong Jin

**Affiliations:** 1 Guangxi Key Laboratory of Green Chemical Materials and Safety Technology, Beibu Gulf University, Qinzhou 535011, China; 2 Guangxi Huayi Energy and Chemical Company Limited, Qinzhou 535008, China

**Keywords:** polybutylene succinate, ball milling, esterified starch, polymer blend

## Abstract

Polybutylene succinate (PBS), as one of the most promising multi-application polymer, still suffers from low toughness, poor miscibility, and high crystallinity. Blending with starch is an effective strategy to improve the properties of PBS, but the compatibility and dispersity between starch and PBS still need to be optimized. In this study, mechanical ball milling was carried out to synthesize esterified starch and the subsequent PBS/esterified starch blend. The FT-IR and XPS analyses confirmed the existence of molecular interactions between PBS and esterified starch. SEM images showed a homogeneous surface for the PBS/esterified starch blend, highlighting the favorable compatibility and good dispersion of starch within the PBS matrix. TGA, DSC, and VSP tests indicated that the introduction of esterified starch into PBS lowered the thermal transition temperatures, thereby enhancing the processability. WCA measurements displayed that the water contact angle of the PBS/esterified starch blends gradually decreased with increasing esterified starch content, proving the improved hydrophilicity of PBS/esterified starch blends. Mechanical testing indicated that incorporating 5 wt% esterified starch into PBS significantly improved the tensile strength to 36.35 ± 2.16 MPa and the breaking elongation to 27.18 ± 5.08%, surpassing those of the pure PBS, PBS/esterified starch mixture, and PBS/starch blend. Our study indicates that mechanical ball milling is an efficient method to improve the properties of PBS composites.

## 1. Introduction

In recent years, the development of sustainable materials has garnered significant attention due to the increasing environmental pollution caused by conventional plastics [1]. Among these materials, polybutylene succinate (PBS) has emerged as one of the most promising sustainable polymers [2,3]. It possesses excellent mechanical properties, good processability, and biodegradability, making it a viable alternative to traditional plastics. However, its practical application is limited by several factors, including high production costs, low toughness, high crystallinity, and poor interfacial adhesion with other polymers. To address these issues, researchers have explored various modification strategies, including blending PBS with natural polymers such as starch.

Starch, one of nature’s most abundant polysaccharides, offers a sustainable and cost-effective solution for enhancing PBS’s properties. However, starch’s inherent hydrophilicity and the poor interfacial adhesion with PBS due to their differing polarities lead to suboptimal blend performances. Esterification of starch has proven to be a highly effective strategy to address these issues [4,5]. It introduces hydrophobic ester groups into starch molecules, enhancing compatibility with PBS and improving the overall properties of the blends. Chemical esterification is a well-established method to modify the chemical structure of starch. Traditional chemical esterification methods involve the use of chemical reagents such as acetic anhydride, maleic anhydride, or octenyl succinic anhydride (OSA). This method, to a certain degree, improves the compatibility of starch with various polymers and enhances the properties of the resulting composites. Despite the above outstanding features, the specific reaction conditions (e.g., controlled pH and temperature [6], catalysts [7], and high temperatures and pressures [8]) of chemical esterification limit its further application.

Mechanical ball milling has recently gained attention as a green and efficient method for esterified starch [9,10]. By applying mechanical force, ball milling can break the ordered crystal structure and reduce the particle size of starch, increase its reactive surface area, and facilitate the esterification reaction. This method not only simplifies the preparation process but also reduces energy consumption compared to traditional chemical methods. In addition, mechanical ball milling can significantly enhance the interfacial adhesion between different polymer components. For instance, Yuan et al. [11] synthesized bacterial cellulose nanocrystal/poly(lactic acid) composite by ball milling. The improved dispersion and enhanced interfacial compatibility of bacterial cellulose nanocrystals in poly(lactic acid) increased the onset degradation temperature and storage modulus of composites. Huang et al. [12] prepared a PBS/carbon nanotube/polytetrafluoroethylene composite through melt mixing under ball milling conditions. The highly dispersed carbon nanotubes and strong interfacial adhesion between polymer components greatly improved the thermal and mechanical properties of the composite.

This study explores the mechanical ball milling-assisted preparation of esterified starch and its subsequent blending with PBS. We aim to investigate how the esterified starch influences the mechanical, thermal, and hydrophilic properties of PBS blends. Additionally, we further study the compatibility between esterified starch and PBS through FT-IR, XPS, and SEM. In this way, our research seeks to provide valuable insights into the structure–property relationships of esterified starch/PBS systems and demonstrate the potential of mechanical ball milling as a scalable and eco-friendly approach for preparing high-performance PBS composites. Therefore, our findings are expected to boost the development of PBS composites and make a positive contribution in packaging, agriculture, and other applications.

## 2. Results and Discussion

### 2.1. Evaluation of Esterified Starch

#### 2.1.1. FT-IR Characterization

**Figure 1:**
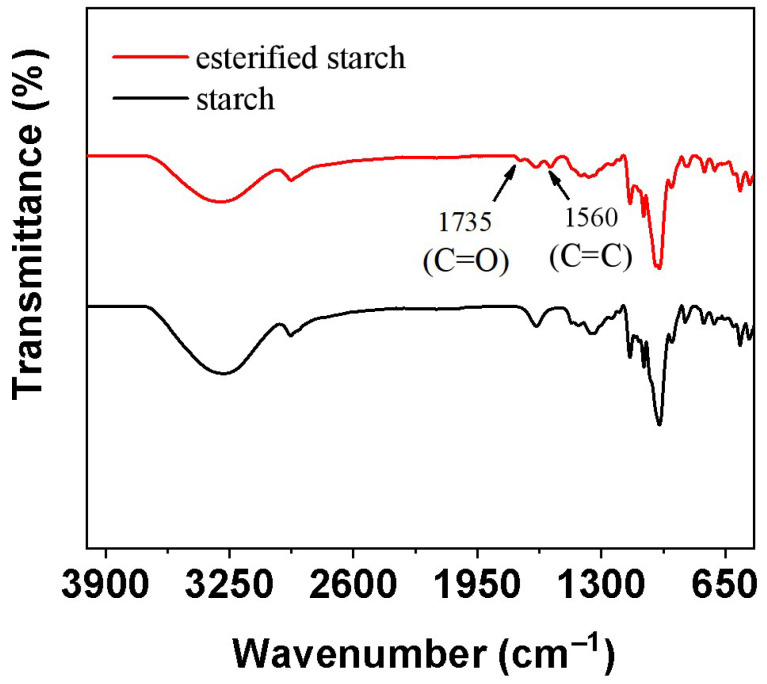
FT-IR of the starch and esterified starch.

FT-IR was employed to analyze the chemical changes in starch before and after esterification via mechanical ball milling (Figure 1). The FT-IR spectra of native starch and esterified starch exhibited significant differences. The native starch showed characteristic absorption peaks at 3400–3200 cm^−1^ (O-H stretching), 2925 cm^−1^ (C-H stretching), and 1077 cm^−1^ (C-O stretching) [13]. After esterification, the O-H stretching peak intensity decreased, indicating a reduction in hydroxyl groups. Simultaneously, a new absorption peak appeared at 1735 cm^−1^ and 1560 cm^−1^, corresponding to the C=O and C=C stretching vibration of ester groups of maleic anhydride [14]. This confirmed the successful esterification of starch. The degree of substitution (DS) of esterified starch increased from 0.1 to 0.2 when extending the ball milling time to 8 h. The formation of ester groups enhanced the interfacial compatibility between starch and PBS, which may enhance the mechanical and biodegradable property of PBS.

#### 2.1.2. XRD Characterization

**Figure 2:**
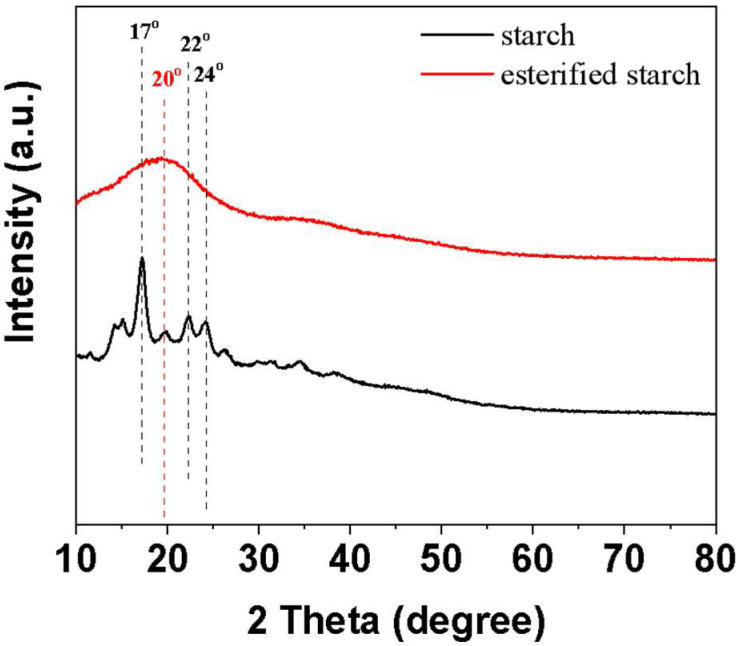
XRD of the starch and esterified starch.

XRD was further utilized to investigate the structural changes in starch and esterified starch (Figure 2). Native starch exhibits characteristic diffraction peaks at approximately 17°, 22°, and 24°, corresponding to its typical crystalline structure. After esterification through mechanical ball milling, the intensity of diffraction peaks decreased, and the positions of diffraction peaks (20°) shifted slightly. Generally, the linear molecular chains tend to regular arrangement, leading to a certain crystallization of native starch [15]. The above results indicate that the crystal structure of linear starch was partially destroyed during the esterification process. The reduction in crystallinity is attributed to the breaking of hydrogen bonds and the rearrangement of starch molecular chains.

#### 2.1.3. TGA Characterization

**Figure 3:**
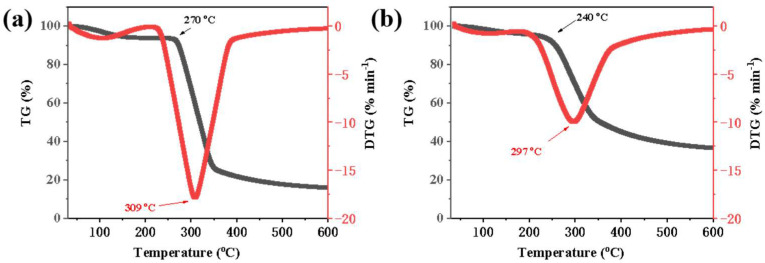
TGA of the (**a**) starch and (**b**) esterified starch at a heating rate of 20 °C min^−1^ under nitrogen flow.

The TGA curves revealed distinct weight loss stages for each material (Figure 3). For native starch, the initial weight loss occurring between 50 and 100 °C was attributed to the removal of free and bound water. The main degradation stage, characterized by a significant weight loss, occurred between 270 and 350 °C due to the thermal degradation of the starch polymer chains [16]. The esterified starch exhibited a lower onset degradation temperature (240 °C vs. 270 °C) and maximum rate of thermal decomposition (297 °C vs. 309 °C) compared to native starch, indicating that the esterification process decreased the thermal stability of starch. This can be attributed to the disruption of the starch crystalline structure during the esterification process, which leads to the esterified starch becoming more susceptible to thermal degradation at lower temperatures.

#### 2.1.4. SEM Characterization

**Figure 4:**
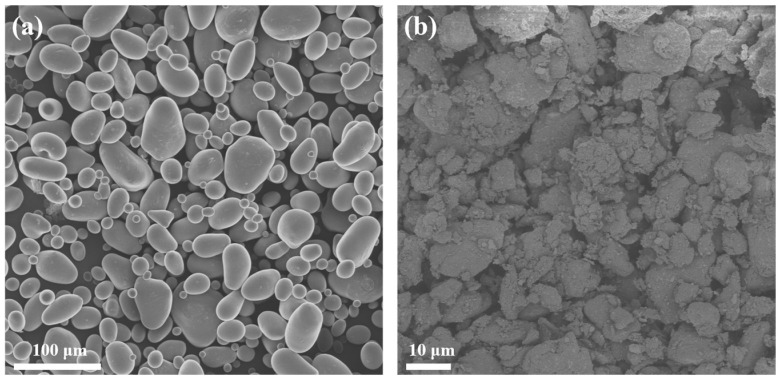
SEM of the (**a**) starch and (**b**) esterified starch.

The morphology of starch and esterified starch was observed through SEM. As shown in Figure 4, native starch exhibits a granular morphology with distinct particle boundaries and a relatively smooth surface. However, esterified starch shows a rougher and irregular surface and smaller particle size after mechanical ball milling and esterification. This transformation can be attributed to the mechanical action of ball milling, which breaks down the starch granules and increases the surface area. The smaller and more rough particles of esterified starch can enhance interfacial compatibility with PBS when blended.

#### 2.1.5. XPS Characterization

**Figure 5:**
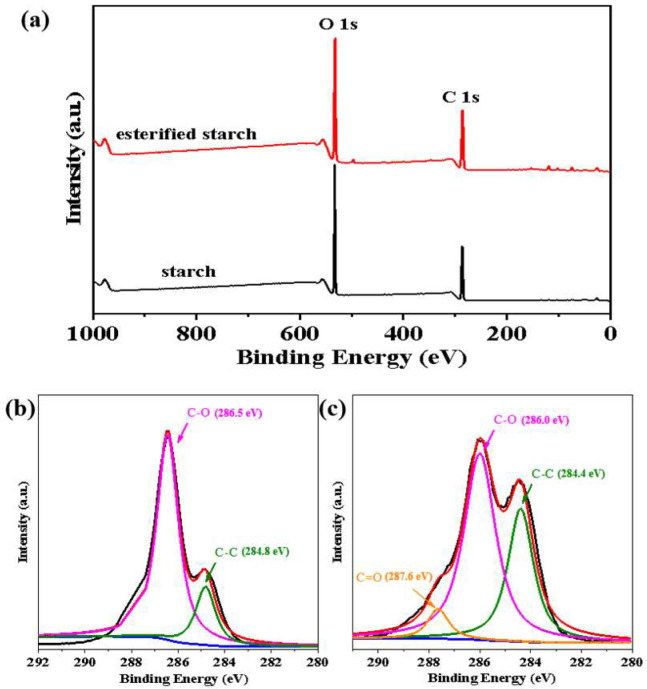
(**a**) XPS survey spectra of the starch and esterified starch. (**b**) C 1s spectrum of the starch. (**c**) C 1s spectrum of the esterified starch.

XPS was employed to analyze the surface chemical composition and chemical states of native starch and esterified starch. The XPS survey spectra showed characteristic peaks at C 1s and O 1s due to the presence of carbon and oxygen elements (Figure 5a). Obviously, the O/C ratio rose from 0.67:1 to 1.15:1 after esterification, suggesting an increase in oxygen-containing functional groups and a reduction in carbonaceous content on the surface of esterified starch. This change confirmed the occurrence of esterification [17]. As shown in Figure 5b, the high-resolution C 1s spectrum of native starch was curve fitted to two peak components, corresponding to a C-C bond (284.8 eV) and C-O bond (286.5 eV). After esterification (Figure 5c), the C 1s spectrum of esterified starch exhibited an additional peak component (C=O) at a higher binding energy (287.6 eV). Moreover, the intensity of the C-C peak increased, while the intensity of the C-O peak decreased compared to native starch. This change was due to the reaction of hydroxyl groups in starch with maleic anhydride, further confirming the successful esterification of starch.

### 2.2. Structure of PBS/Esterified Starch Blend

#### 2.2.1. Chemical Structure of PBS/Esterified Starch Blend

**Figure 6:**
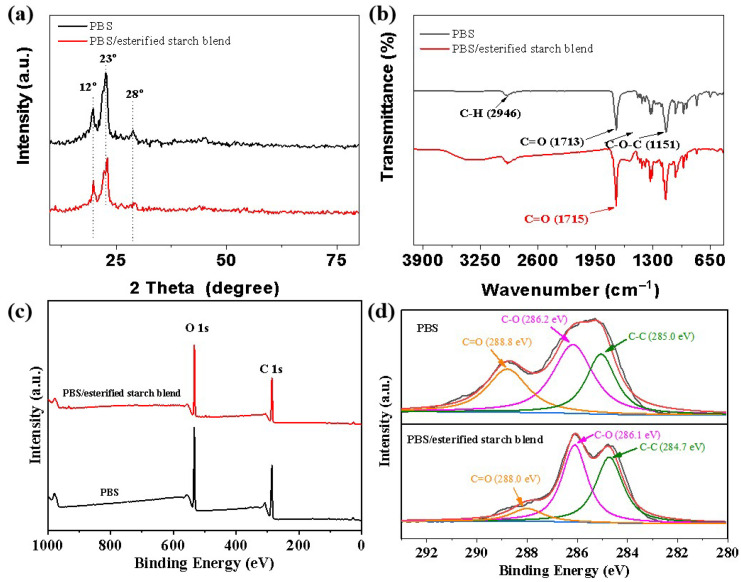
(**a**) XRD patterns of the pure PBS and PBS/esterified starch blend. (**b**) FT-IR spectra of the pure PBS and PBS/esterified starch blend. (**c**) XPS survey spectra of the pure PBS and PBS/esterified starch blend. (**d**) C 1s spectra of the pure PBS and PBS/esterified starch blend.

The crystallinity and crystalline structures of pure PBS and the PBS/esterified starch blend are shown in Figure 6a. Pure PBS exhibited distinct diffraction peaks at approximately 12°, 23°, and 28°, corresponding to its characteristic crystalline planes. Notably, the intensity of the characteristic peaks of PBS decreased in the PBS/esterified starch blend, indicating a reduction in crystallinity due to the incorporation of amorphous esterified starch. Moreover, the diffraction peaks of the PBS/esterified starch blend shifted slightly compared to that of pure PBS, indicating an enhanced interaction between PBS and esterified starch [18]. Based on this result, the introduction of esterified starch not only enhances the interfacial compatibility of the PBS but also improves the thermoplastic property of the blend.

The FT-IR spectra of pure PBS exhibited characteristic absorption peaks at 1713 cm^−1^ (C=O stretching in ester groups), 1151 cm^−1^ (C-O-C stretching), and 2946 cm^−1^ (C-H stretching), corresponding to the typical ester bonds and aliphatic chain in PBS. In the PBS/esterified starch blend, the characteristic peaks of both components were present, indicating good miscibility (Figure 6b). Moreover, the C=O stretching peak of PBS shifted slightly to a higher wavenumber, suggesting hydrogen bonding interactions between the ester groups of esterified starch and the ester bonds in PBS [19].

XPS analysis was further conducted to investigate the surface chemical composition and electronic states of pure PBS and the PBS/esterified starch blend. The XPS survey spectra of pure PBS and PBS/esterified starch showed characteristic peaks at C 1s and O 1s (Figure 6c). As shown in Table 1, the O/C ratio increased after the addition of esterified starch, suggesting an increase in oxygen-containing functional groups, which confirmed the successful incorporation of esterified starch into the PBS matrix. The C 1s core-level spectrum of pure PBS was curve fitted to three peak components corresponding to a C-C bond (285.0 eV), C-O bond (286.2 eV), and C=O bond (288.8 eV). In the PBS/esterified starch blend (Figure 6d), the intensity of the C=O peak decreased, while the intensity of the C-O peak increased compared to pure PBS, indicating a growth in hydroxyl content. Moreover, the C=O peak position of PBS/esterified starch (288.0 eV) showed a big change compared to those of pure PBS and esterified starch, which may be caused by the hydrogen bonding interactions between esterified starch and PBS [20].

#### 2.2.2. Morphology of PBS/Esterified Starch Blend

**Figure 7:**
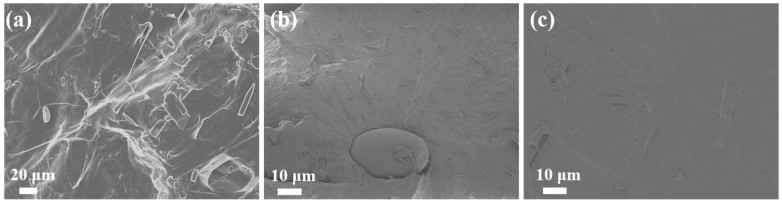
SEM of the (**a**) PBS, (**b**) PBS/starch blend, and (**c**) PBS/esterified starch blend.

The SEM images of pure PBS and different types of PBS/esterified starch blends are presented in Figure 7. Pure PBS revealed a relatively smooth and uniform surface. For the PBS/starch blend, the large starch particle embedded into the PBS matrix with sharp boundaries, indicating weak interfacial adhesion between PBS and pure starch. This poor compatibility is attributed to the hydrophilic nature of starch and the hydrophobic nature of PBS, leading to phase separation and potential structure failure in the blend. In contrast, the PBS/esterified starch blend displayed a more homogeneous surface morphology, indicating a good dispersion of esterified starch particles in the PBS matrix. This result indicates that esterification of starch and the mechanical action of ball milling can improve the interfacial compatibility between starch and PBS. The enhanced interfacial adhesion can significantly enhance the mechanical properties of the blend by facilitating effective stress transfer between the two phases.

### 2.3. Thermal Performance of PBS/Esterified Starch Blend

**Figure 8:**
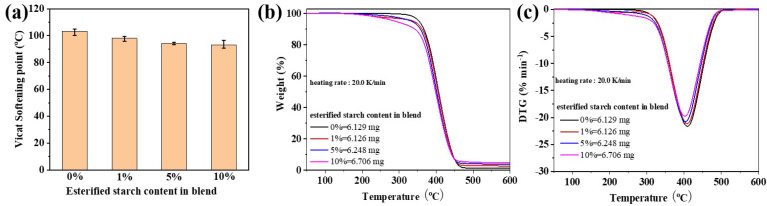
(**a**) Vicat softening temperature of the PBS/esterified starch blend with different esterified starch contents at a heating rate of 120 °C h^−1^. (**b**) TGA curves and (**c**) DTG curves of the PBS/esterified starch blend with different esterified starch contents at a heating rate of 20 °C min^−1^ under nitrogen flow.

Figure 8a showed the Vicat softening temperature of the PBS/esterified starch blend with different esterified starch contents. The Vicat softening temperature of the PBS/esterified starch blend revealed a trend of decrease with the increase in esterified starch content. The Vicat softening temperature of PBS decreased from 103.3 ± 1.7 °C to 93.2 ± 2.5 °C after the introduction of 10% esterified starch. The reason was that the addition of amorphous esterified starch leads to a decrease in the crystallinity of the PBS/esterified starch blend, resulting in the reduction in Vicat softening temperature. Figure 8b shows the TGA curves and related DTG curves of the blends. Pure PBS exhibited a single-stage weight loss in the temperature range of 340–460 °C, corresponding to its thermal degradation. The initial decomposition temperature (onset temperature) was approximately 340 °C, and the maximum decomposition temperature was around 409 °C. Interestingly, the PBS/esterified starch blend showed a two-stage weight loss in the TGA curves. The first stage belongs to the pyrolysis of esterified starch, and the second stage corresponds to the thermal degradation of the polymer matrix. Moreover, it can be seen that with increasing esterified starch content, the maximum decomposition temperature of the PBS/esterified starch blend decreased, and the residual weight at 600 °C increased. One of the reasons was that the interfacial interactions between PBS and esterified starch enhanced thermal stability at higher temperatures. The other reason was that the introduction of amorphous esterified starch caused a decrease in the maximum decomposition temperature.

### 2.4. Differential Scanning Calorimetry (DSC) Analysis of PBS/Esterified Starch Blend

**Figure 9:**
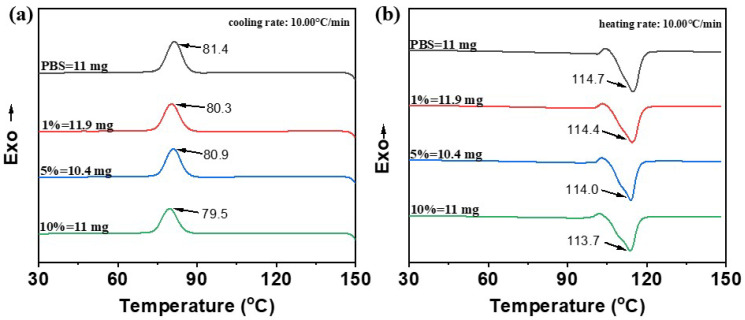
DSC curves of PBS and PBS/esterified starch (1%, 5% and 10%) blends. (**a**) Cooling curve; (**b**) heating curve at a temperature change rate of 10 °C min^−1^.

The DSC curves of pure PBS revealed a crystallization temperature (*T*_c_) at approximately 81.4 °C and a melting temperature (*T*_m_) around 114.7 °C (Figure 9). The addition of esterified starch led to a slightly decrease in these thermal transition temperatures. The *T*_c_ and *T*_m_ of the blends decreased slightly with esterified starch content, reaching a minimum of 79.5 °C for *T*_c_ and 113.7 °C for *T*_m_ at 10% esterified starch. This reduction in both *T*_c_ and *T*_m_ reveals that the addition of esterified starch makes the PBS/esterified starch blend easier to crystallize, leading to an enhancement of processability. The degree of crystallinity (*X*_c_) was calculated through the melting enthalpy, and the results indicated that the crystallinity of the blends greatly decreased after addition of esterified starch, suggesting a strong disruption of the PBS crystalline structure (Table 2).

### 2.5. Water Contact Angle (WCA) of PBS/Esterified Starch Blend

**Figure 10:**
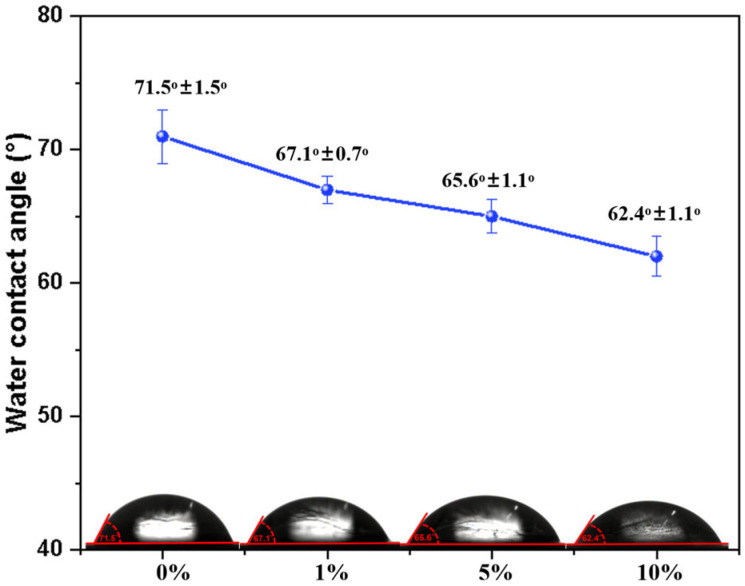
WCA of PBS/esterified starch blend with different esterified starch contents.

The hydrophilicity of PBS and the PBS/esterified starch blend was evaluated via water contact angle measurements. It is believed that the water absorption effect of esterified starch can greatly influence the hydrophilicity of PBS. As shown in Figure 10, pure PBS exhibited a relatively high water contact angle of 71°, which can be attributed to the crystalline structure and the hydrophobic segments in PBS. After incorporation of esterified starch into PBS, the WCA of the blends decreased. The order of WCA was PBS/esterified starch (10%) blend (62.4° ± 1.1°) < PBS/esterified starch (5%) blend (65.6° ± 1.1°) < PBS/esterified starch (1%) blend (67.1° ± 0.7°) < PBS (71.5° ± 1.5°). This result indicates that the PBS/esterified starch blend becomes more hydrophilic as the esterified starch content increases. After esterification, partly hydroxyl groups on the polymer chain of starch were replaced by ester groups, revealing an increasing WCA of esterified starch compared to starch [21]. The good dispersion and hydrogen bonding interactions between esterified starch and PBS weakened the functionality of ester groups and enhanced the transfer of water molecules between hydroxyl groups, leading to a decreased WCA of the PBS/esterified starch blend.

### 2.6. Mechanical Properties of PBS/Esterified Starch Blend

**Figure 11:**
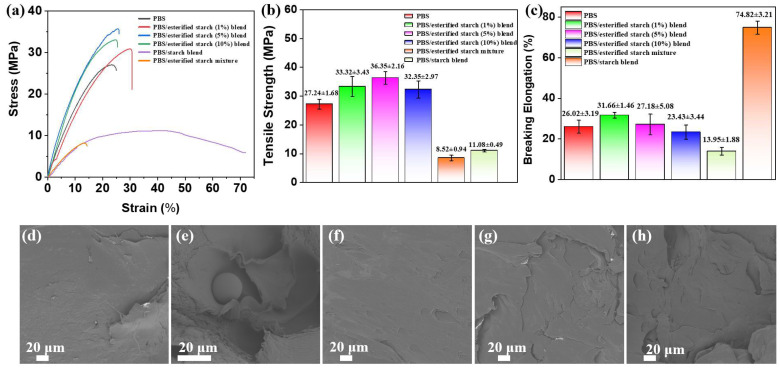
(**a**) Stress–strain curves, (**b**) tensile strength, and (**c**) breaking elongation of PBS/esterified starch (0%, 1%, 5%, and 10%) blend, PBS/starch blend, and PBS/esterified starch mixture (five tensile tests for each sample). SEM images of the fracture surfaces of (**d**) PBS, (**e**) PBS/starch blend, (**f**) PBS/esterified starch (1%) blend, (**g**) PBS/esterified starch blend, and (**h**) PBS/esterified starch (10%) blend.

The mechanical properties of the PBS/esterified starch blend were evaluated through tensile testing. As shown in Figure 11a, the PBS/esterified starch blend showed increased tensile stress and strain compared to pure PBS and revealed brittle fracture behavior without yield point. Pure PBS exhibited a tensile strength of approximately 27.24 ± 1.68 MPa. Upon incorporation of starch, the tensile strength greatly decreased to 11.08 ± 0.49 MPa, indicating poor mechanical property of the PBS/starch blend. In contrast, the PBS/esterified starch blend with 1 wt% esterified starch showed a tensile strength of 33.32 ± 3.43 MPa, representing a 22% improvement over pure PBS (Figure 11b). This enhancement is attributed to the strong interfacial adhesion and homogeneous dispersion between the esterified starch and PBS matrix. The well-dispersed esterified starch can effectively transfer stress and enhance the load-bearing capacity of the blend. As the esterified starch content increased to 5 wt%, the tensile strength slightly further increased to 36.35 ± 2.16 MPa. Moreover, the PBS/esterified starch mixture without ball milling showed a relatively low tensile strength (8.52 ± 0.94 MPa), which indicates the important role of ball milling in improving the properties of PBS composites. Further increases in esterified starch content (10 wt%) led to a decline in tensile strength. This trend suggests that excessive esterified starch content may lead to phase separation, thereby reducing the reinforcing effect. In addition, the elongation at break was also evaluated (Figure 11c). Pure PBS demonstrated a breaking elongation of approximately 26.02 ± 3.19%. With the addition of 1 wt% esterified starch, the breaking elongation slightly increased to 31.66 ± 1.46%. With the esterified starch content increased to 10 wt%, the breaking elongation dropped to 23.43 ± 3.44%. This reduction highlights the brittleness introduced by higher esterified starch concentrations. The reason was that the higher esterified starch contents can decrease the crystallinity of the PBS/esterified starch blend, leading to a more rigid and less flexible structure. SEM images of the fracture surfaces of the PBS/esterified starch blend were carried out to investigate the morphological changes and interfacial interactions within the materials (Figure 11d–h). Pure PBS exhibits a relatively smooth and regular fracture surface. The presence of starch resulted in a spherical particle and numerous holes shown on the sample surface, indicating a poor interfacial compatibility. For the PBS/esterified starch (1% and 5%) blend, a homogeneous surface was shown compared to that of the PBS/starch blend, revealing a good interface adhesion between PBS and esterified starch [22,23]. This good interface adhesion facilitates effective stress transfer between the two phases, leading to enhanced mechanical properties. However, a certain degree of phase separation occurred when the esterified starch content increased to 10 wt%, which can reduce the efficiency of stress transfer and lead to a reduction in tensile strength. 

## 3. Materials and Methods

### 3.1. Materials

Polybutylene succinate (PBS) was purchased from PTT Public Company Limited (Bangkok, Thailand) with a molecular weight of 8.5 × 10^4^ g mol^−1^ (obtained from Agilent 1260 Infinity II HT GPC SYSTEM, Santa Clara, CA, USA). Other reagents such as starch, sulfuric acid, maleic anhydride, and anhydrous ethanol were acquired from Aladdin (Shanghai, China) and used without further purification.

### 3.2. Preparation of Samples

#### 3.2.1. Preparation of Esterified Starch

Esterified starch was synthesized through an esterification reaction under mechanical ball milling. Here, 1.4 g of starch, 0.7 g of maleic anhydride, and 1 mL of sulfuric acid were mixed and added into the ball mill tank. After ball milling for 8 h, the mixture was washed with anhydrous ethanol and vacuum dried to obtain the esterified starch. For comparison, the different ball milling time (4 h) was carried out.

#### 3.2.2. Preparation of PBS/Esterified Starch Blend

To obtain the PBS/esterified starch blend, PBS and esterified starch in a weight ratio of 95:5 were blended using a mechanical ball milling process. The blend was subjected to ball milling at room temperature for 8 h at 400 rpm to ensure thorough mixing and dispersion. For comparison, the PBS/esterified starch blend in different ratios and the PBS/starch blend were prepared through the same procedure. The PBS/esterified starch mixture was synthesized by mixing PBS and esterified starch without ball milling.

The detail ball milling procedure was operated as follows: The raw materials were added to a 250 mL zirconium oxide jar along with 25 zirconium balls of 12 mm diameter and 86 small zirconium balls of 6 mm diameter (ball-to-powder mass ratio of 25:1). Then, a planetary ball mill was employed: the jar was initially counter-rotated for 240 min and then stopped for 10 min, followed by forward rotation for another 240 min to obtain the product.

### 3.3. Characterization Techniques

The characterization data were analyzed by Origin 2018 software.

#### 3.3.1. Fourier Transform Infrared (FT-IR)

To investigate the functional groups, FT-IR (Perkin Elmer Frontier, Waltham, MA, USA) was detected in transmission mode using a (Perkin Elmer Frontier) spectrometer in the range of 500 to 4000 cm^−1^.

#### 3.3.2. Powder X-Ray Diffraction (XRD)

XRD patterns were studied using a XRD instrument (D8 Advance, Bruker, Billerica, MA, USA) operating at 40 kV using Cu Kα radiation (λ = 0.1541 Å) with a range from 10° to 80° at a rate of 10° min^−1^.

#### 3.3.3. X-Ray Photoelectron Spectroscopy (XPS)

XPS analysis was performed on a Thermo Fisher Scientific K-Alpha spectrometer (Thermo Fisher Scientific, Waltham, MA, USA) to determine surface elemental composition and chemical states.

#### 3.3.4. Scanning Electron Microscopy (SEM)

Morphological analysis of the samples was carried out using an Apreo 2SHiVac field-emission scanning electron microscope (Thermo Fisher Scientific, USA). Samples were sputter coated with gold prior to observation at an accelerating voltage of 5 kV.

#### 3.3.5. Water Contact Angle (WCA) Measurements

A JC200D2 contact angle goniometer (Zhongchen Digital Technology Equipment (Shanghai) Co., Ltd., Shanghai, China) was used to measure the water contact angle to evaluate the hydrophilicity of the samples. Distilled water was used as the test liquid, and measurements were taken at five different locations on each sample.

#### 3.3.6. Thermogravimetric Analysis (TGA)

Thermal stability was assessed using a NETZSCH STA 2500 thermogravimetric analyzer (NETZSCH, Selb, Germany). Samples were heated from 30 °C to 800 °C at a rate of 20 °C min^−1^ under nitrogen flow.

#### 3.3.7. Vicat Softening Point Temperature (VSP)

Vicat softening point temperature was detected using a KL-WK-300F machine (Kunlun Testing Instruments (Dongguan) Co., Ltd., Dongguan, China). The test was conducted in accordance with GB/T 1633-2000 [24], the load weight was 10 N, and the heating rate was 120 °C h^−1^.

#### 3.3.8. Differential Scanning Calorimetry (DSC)

Differential Scanning Calorimetry (DSC) analysis was performed on a Discovery DSC25 Calorimeter (TA instruments, New Castle, DE, USA) in accordance with GB/T19466.3-2004 [25] under a nitrogen atmosphere. For typical procedure, the sample with a mass range of 5–8 mg was first heated to 150 °C and held at 150 °C for 5 min. Subsequently, the temperature was reduced to −50 °C, held for 5 min, and then reheated to 150 °C. The variation in heat enthalpy (Δ*H*) over time (*t*) was monitored during heating, with both heating and cooling rates maintained at 10 °C min^−1^.

The crystallinity of the samples is calculated using Equation (1):

(1) $$Xc=\frac{{\Delta H}_{m}}{\Delta H\omega_{PBS}}\times 100\%$$

where Δ*H*_m_ is the melting enthalpy of the sample, Δ*H* is the theoretical melting enthalpy of the completely crystallized PBS (the Δ*H* of PBS is about 110.3 J g^−1^), and *w*_PBS_ is the mass fraction of PBS in the blends.

#### 3.3.9. Mechanical Testing

To prepare the dumbbell-shaped specimens for mechanical testing, 100 g of the PBS/esterified starch blend and 6 g of glycerin was first mixed at 130 °C using an open mill. After that, the mixture was preheated in a hot press at 135 °C for 3 min and kept at a constant pressure of 10 MPa for 10 min. Finally, the dumbbell-shaped specimens were obtained after being pressed at room temperature for 5 min and dried to unchanged weight. Tensile properties were evaluated using an HZ-1004A universal testing machine (Lixian Instrument Technology (Dongguan) Co., Ltd., Dongguan, China) according to GB/T 1040-2018 [26] and ISO 527-1:2019 [27]. Dumbbell-shaped specimens were tested at room temperature at a crosshead speed of 2 mm min^−1^. Five tensile tests were applied to each sample, and the average values were calculated to minimize errors.

#### 3.3.10. Degree-of-Substitution (DS) Experiment

According to the DS method by Li et al. [28], 0.4 g of sample, 5 mL of deionized water, and 2 mL of 0.2 mol L^−1^ NaOH solution were mixed in the 50 mL flask and shaken at 37 °C for 50 min to form a homogeneous solution. After adding 2 drops of 0.1% phenolphthalein indicator, the mixture was titrated with 0.1 mol L^−1^ HCl solution until the red color disappeared and remained colorless for 10 s. The volume of HCl solution consumed in the titration was recorded.

The DS value was calculated using the following equation:

(2) $$DS=\frac{162c(v_{0}-v_{1})}{1000m-Mc(v_{0}-v_{1})}$$

where *DS* is the degree of substitution; *c* is the molar concentration of the hydrochloric acid standard solution (mol L^−1^); m is the mass of esterified starch (g); *M* is the molecular weight of the maleic anhydride acyl group; *V*_0_ is the volume of the standard hydrochloric acid solution consumed for the control sample during titration (mL); and *V*_1_ is the volume of the standard hydrochloric acid solution consumed for the esterified starch during titration (mL).

## 4. Conclusions

In this paper, mechanical ball milling was carried out to prepare esterified starch and an PBS/esterified starch blend. The mechanical ball milling process effectively introduced hydrophobic ester groups into starch molecules and simultaneously improved its compatibility with PBS, leading to an enhanced performance of the PBS/esterified starch blend. The key findings are summarized as follows:

SEM images showed a homogeneous surface morphology of the PBS/esterified starch blend, indicating that mechanical ball milling favored the construction of favorable interfacial compatibility between PBS and the esterified starch.

TGA, VSP, and DSC revealed that the PBS/esterified starch blend displays a decrease in the maximum decomposition temperature and thermal transition temperatures with increasing esterified starch content, making the blend more processable.

WCA measurements demonstrated that the hydrophilicity of the PBS/esterified starch blend decreased as the esterified starch content increased.

In mechanical tests, all ratios of the PBS/esterified starch blend displayed higher tensile strength compared to pure PBS. Among them, the blend with 5 wt% esterified starch exhibited the highest tensile strength of 36.35 ± 2.16 MPa and an elongation at break of 27.18 ± 5.08%.

In the end, mechanical ball milling has been proven as an efficient method for improving the properties and compatibility of the PBS/esterified starch blend, which will provide valuable solutions in developing high-performance PBS composites for packaging, agriculture, and other applications.

## Figures and Tables

**Table 1:** Element contents of the samples.

Samples	C Content (at%)	O Content (at%)
starch	59.94	40.06
esterified starch	46.58	53.42
PBS	64.47	35.53
PBS/esterified starch blend	59.16	40.84

**Table 2:** DSC data of PBS and PBS/esterified starch (1%, 5%, and 10%) blends.

Samples	*T*_c_/°C	*T*_m_/°C	Δ*H*_c_/J g^−1^	Δ*H*_m_/J g^−1^	*X*_c_/%
PBS	81.4	114.7	66.8	70.1	63.55
PBS/esterified starch (1%) blends	80.3	114.4	60.4	57.7	52.84
PBS/esterified starch (5%) blends	80.9	114.0	59.0	55.0	52.49
PBS/esterified starch (10%) blends	79.5	113.7	54.1	52.4	52.79

## Data Availability

The original contributions presented in the study are included in the article; further inquiries can be directed to the corresponding authors.

## Abbreviations

**Abbreviation**	**Expression**
PBS	Poly butylene Succinate
DS	Degree of Substitution
FT-IR	Fourier Transform Infrared Spectroscopy
XPS	X-ray Photoelectron Spectroscopy
SEM	Scanning Electron Microscope
TGA	Thermogravimetry Analysis
WCA	Water Contact Angle
XRD	X-ray Diffraction
VSP	Vicat Softening Point Temperature
